# Population Structure of the Rockpool Blenny *Entomacrodus vomerinus* Shows Source-Sink Dynamics among Ecoregions in the Tropical Southwestern Atlantic

**DOI:** 10.1371/journal.pone.0157472

**Published:** 2016-06-16

**Authors:** Jessika M. M. Neves, Sergio M. Q. Lima, Liana F. Mendes, Rodrigo A. Torres, Ricardo J. Pereira, Tamí Mott

**Affiliations:** 1 Laboratório de Diversidade Molecular, Setor de Biodiversidade, Universidade Federal de Alagoas, Maceió, Alagoas, Brazil; 2 Laboratório de Ictiologia Sistemática e Evolutiva, Departamento de Botânica e Zoologia, Universidade Federal do Rio Grande do Norte, Natal, Rio Grande do Norte, Brazil; 3 Laboratório do Oceano, Departamento de Ecologia, Universidade Federal do Rio Grande do Norte, Natal, Rio Grande do Norte, Brazil; 4 Laboratório de Genômica Evolutiva e Ambiental, Departamento de Zoologia, Universidade Federal de Pernambuco, Recife, Pernambuco, Brazil; 5 Centre for GeoGenetics, Natural History Museum of Denmark, University of Copenhagen, Øster Voldgade, Copenhagen, Denmark; Ecole normale superieure de Lyon, FRANCE

## Abstract

The Tropical Southwestern Atlantic is characterized by prominent ecosystems with large-scale oceanographic complexity. Yet, the evolutionary processes underlying genetic differentiation and connectivity in this region remain largely unknown. *Entomacrodus vomerinus* (Valenciennes, 1836) is a demersal fish with planktonic larvae endemic to this marine province, inhabiting shallow tidal pools in continental and oceanic reef environments. We evaluated the population structure, genetic diversity and gene flow of *E*. *vomerinus* using mitochondrial data (CYTB and COI) and nuclear (rhodopsin, RHO) DNA sequences. We sampled a total of 85 individuals, comprising 46 from three oceanic archipelagos with varying distance from the coast (São Pedro and São Paulo—SS, Fernando de Noronha—FE and Rocas Atoll—RA) and 39 from two localities in northeastern Brazilian coast (Rio Grande do Norte—RN and Bahia—BA). Multilocus analysis revealed the presence of three Evolutionarily Significant Units—ESUs (SS, FE+RA, and RN+BA), which are in accordance with distinct marine ecoregions. Coalescent analyses showed that the central ESU has a larger effective population size than the other two, suggesting strong asymmetries in the genetic diversity across the species range. Moreover, they showed that gene flow is highly asymmetric, suggesting a source-sink dynamics from the central ESU into the remaining ones, in agreement with oceanic currents. Together, these results provide insights in the evolutionary mechanisms facilitating diversification in this marine province.

## Introduction

Phylogeographic studies seek to reveal biogeographical history of species and the habitats they occupy via the spatial association between clusters of alleles with geographic/ecological barriers, and via estimates of historical population size [1,2]. A more detailed record of historical divergence and demography might be preserved in the genetic patterns of taxa showing finer spatial scales of persistence and low vagility [3,4]. In marine habitats, species whose adults have sedentary habits and small home range, displaying reduced dispersal ability over long distances [5,6], are good candidates for recovering details of historical biogeography.

Many studies of marine species assume that the presence of planktonic larval period gives a greater dispersal ability, increasing gene flow between geographically isolated populations and hence reducing the population structure. However, a meta-analysis involving molecular data of marine organisms from 300 studies suggests that a long larval period is not a good predictor of gene flow between populations [7]. Thus, it is essential to use molecular tools to uncover phylogeographic patterns of population isolation and connectivity, particularly in species were such processes are obscured by cryptic morphologic divergence. Hence, molecular techniques provide indirect information about larval dispersion and population connectivity, overcoming the problems behind larval developmental stages and spatial complexity of marine ecosystems [8]. Genetic analysis can also detect levels of migration, isolation, drift and selection within and among populations [9], being of fundamental importance also in the delimitation of Evolutionarily Significant Units (ESUs) for management and conservation purposes [10].

The rockpool blenny *Entomacrodus vomerinus* (Valenciennes, 1836) is endemic to the Tropical Southwestern Atlantic province (*sensu* [11]), inhabiting four of the five ecoregions [12], including two coastal (Northeastern Brazil, and Eastern Brazil) and two insular (São Pedro and São Paulo Islands; Fernando de Noronha and Rocas Atoll). The fifth oceanic ecoregion (Trindade and Martin Vaz Islands) is inhabited by an endemic closely related species [13,14]. As other congeneric species, *E*. *vomerinus* has pelagic larvae of unknown duration, the adults have a gregarious (group of three up to 40 individuals) and sedentary behavior, almost exclusively inhabiting shallow tidal pools [12,13], with small home ranges of near 2 x 2 m^2^ [15]. Moreover, adults have amphibious habits, being observed up to 20 minutes out of the water [12,15]. This species is considered as “least concern” by the International Union for Conservation of Nature (IUCN) [16]. Nevertheless, there is no current knowledge whether such species correspond to a single or multiple ESUs, if genetic diversity is partitioned evenly throughout the species’ range, or if populations are connected by gene flow. Moreover, because the adult stage of this species is restricted to tidal pools that get dammed during low tides, local populations can be easily affected by anthropogenic activities such as pollution of the coastline [16].

Springer (1972) found differences in the number of dorsal rays between *E*. *vomerinus* specimens from the Brazilian coast and the oceanic islands (Fernando de Noronha and São Pedro and São Paulo) [13]. It remains unclear whether these coast and island groups of *E*. *vomerinus* correspond to different evolutionary units, and the use of DNA techniques were suggested to address this question [13]. Thus, our null hypothesis is that *E*. *vomerinus* populations are genetically structured in agreement with morphological variation. Such genetic subdivision in *E*. *vomerinus* could be expected because insular and coastal environments are potentially different in terms of adaptive boundaries [11,17]. Finer genetic subdivision may also occur if the marine ecoregions inhabited by this species coincide with important geographic or ecological barriers. This has been shown in some marine organisms from Tropical Southwestern Atlantic inhabiting coastal [18,19], island environments [20], or both [21]. Nonetheless, the patterns and processes underlying the genetic distribution of marine lineages in South America are largely unknown, and could be important to understand macroecology, impacts of geological features in species diversification and to detect areas of high conservation priority [22].

The main goal of this study was to investigate the phylogeographic pattern of *Entomacrodus vomerinus* among shallow reef coastal and insular environments in the Tropical Southwestern Atlantic. The specific goals were: (1) to assess the levels of genetic diversity and connectivity of *E*. *vomerinus* throughout this province, (2) to investigate how possible phylogeographic breaks and dispersal corridors are related to oceanographic features, and (3) to define possible ESUs, suggesting conservation priorities in some marine protected areas (MPAs).

## Materials and Methods

### Sampling

**Fig 1:**
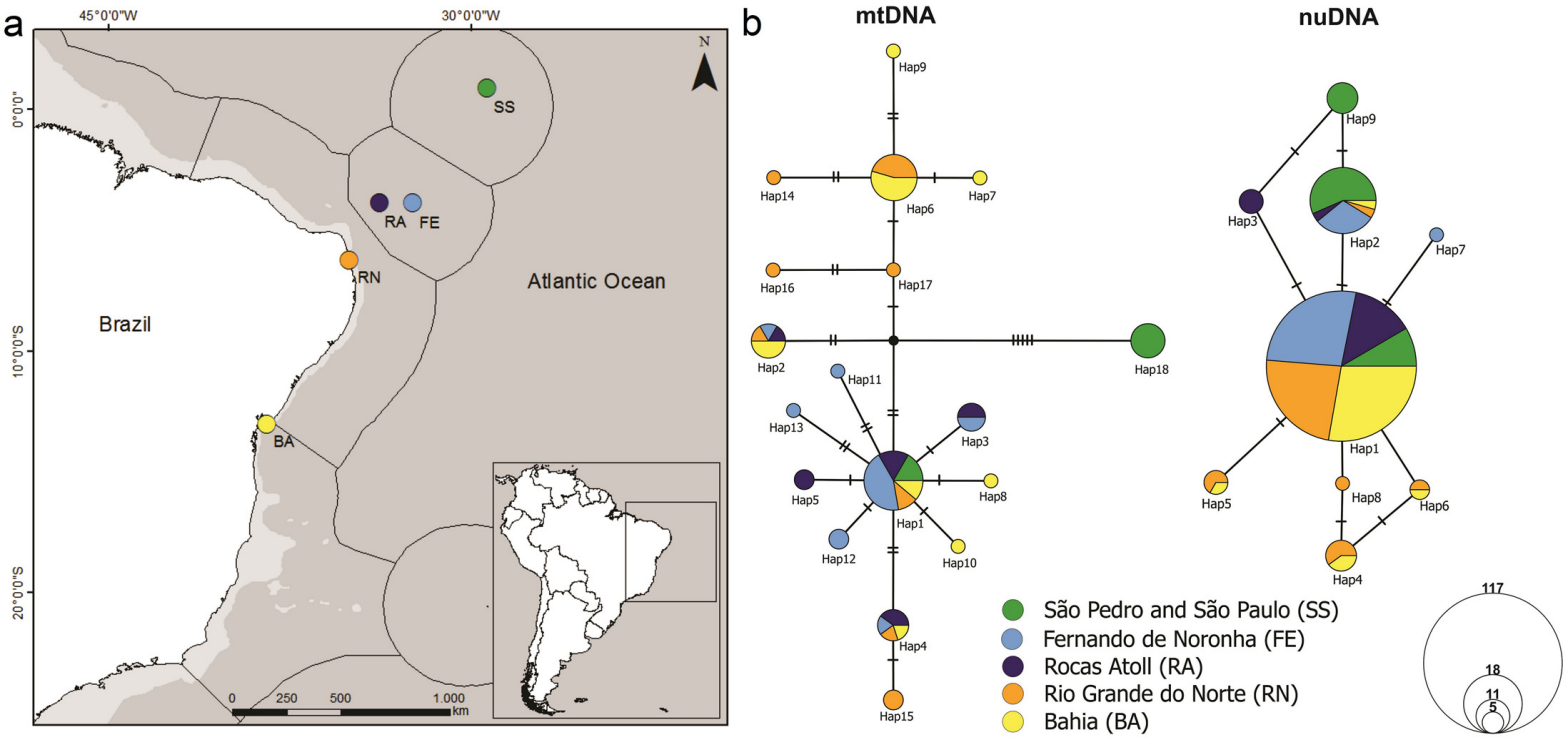
a Sampling sites of *Entomacrodus vomerinus* in the Brazilian oceanic islands and coast, with marine ecoregions of the Tropical Southwestern Atlantic province and inset map of South America. b Haplotype networks from TCS analysis, using 95% probability of parsimony, of mtDNA (COI and CYTB) and nuDNA (RHO) with each haplotype represented by a circle size proportional to its frequency.

In total, eighty-five individuals of *Entomacrodus vomerinus* were collected throughout the entire species' distribution, encompassing the four ecoregions of the Tropical Southwestern Atlantic province inhabited by this species (Fig 1a). Forty-six individuals are from insular localities: 16 from the São Pedro and São Paulo Islands ecoregion [SS] (0°55' 00.7"N 29°20' 44.9" W), 30 from Fernando de Noronha [FE] (3°50'38.5"S 32°25'43.3"W) and Rocas Atoll [RA] (3°52'19.2"S 33°47'52.6"W) ecoregion [20 from FE and 10 from RA]. Thirty-nine individuals are from continental environments: 18 from northeastern Brazil ecoregion (Tibau do Sul Municipality, south of Rio Grande do Norte State [RN], 6°13'37.8"S 35°03'05.2"W), and 21 from eastern Brazil ecoregion (Salvador Municipality, Bahia State [BA], at two localities (13°00'40.8"S 38°30'32.1"W and 13°00'14.8"S 38°32'01.7"W). This last sampling site is the species’ type locality and is situated in the border of both coastal ecoregions.

All specimens were collected at daytime in tidal pools. Adult individuals were captured using a transparent plastic bags arranged along the reefs. After collecting, individuals were anesthetized and euthanized with clove oil (eugenol), stored in absolute alcohol and deposited in the ichthyological collection of the Universidade Federal do Rio Grande do Norte (UFRN) (S1 Table). Coastal samples were obtained under MMA/ICMBio/SISBIO #30532/2011 permit, while oceanic islands ones using MMA/ICMBio/SISBIO #10806/2011, issued by Ministério Brasileiro do Meio Ambiente/Instituto Chico Mendes de Conservação da Biodiversidade/Sistema de Autorização e Informação em Biodiversidade.

### Molecular Markers

Total genomic DNA extraction followed the phenol/chloroform protocol [23].

Fragments from two mitochondrial genes (Cytochrome C Oxidase subunit I [COI] and Cytochrome B [CYTB]) and a nuclear gene (the exon Rhodopsin [RHO]) were amplified by polymerase chain reactions (PCR). The COI fragment was amplified using the primers FISH-BCL [24] and FISH-BCH [25]. The RHO fragment was amplified using the primers F2w-Rod and Rod-R4n [26]. For both genes, a 25 μl PCR reaction included: 0.8–2 μl of genomic DNA (20–80 ng/μl), 12.5 μl of PCR Master Mix (Promega^®^), 1 μL of each primer (10 mM), 0.5 μl of 1 U Taq Polymerase and ultrapure water. The amplification followed an initial DNA denaturation at 95°C for 5 min, then 35 cycles of denaturation at 94°C for 30 s, annealing at 52°C for 35 s, extension at 72°C for 1 min, followed by a final extension at 72°C for 7 min and 20°C for 2 min. The CYTB fragment was amplified using the primers L14725 (CGAAACTAATGACTTGAAAAACCACCGTTG) (Sampaio, unpublished) and HMVZ16 [27]. A 25 μl PCR reaction included: 1 μL of genomic DNA (20–80 ng/μl), 12.5 μl Taq Master Mix (Vivantis^®^), 0.5 μl of each primer (10 mM), 0.3 μl de MgCl_2_ (3 mM) and 10.2 μl of ultrapure water. Amplification followed the protocol established [28]. All PCR reactions with amplicons were purified by the isopropanol method and resuspended in 15 μl of ultrapure water.

After purification, the amplicons were sequenced using forward primers in the Sequencing facility from Centro de Biociências, Universidade Federal de Pernambuco. RHO sequences that showed heterozygous sites were additionally sequenced with the reverse primer to confirm polymorphisms.

### Sequence alignments

All DNA sequences were edited and aligned using Bioedit 7.2 [29]. Subsequently codons were translated into amino acids at MEGA6 [30] to verify the absence stop codons caused by editing errors.

Mitochondrial genes (COI and CYTB) were concatenated and incorporated as a single non-recombining marker in all analyses (mtDNA hereafter). Due to the presence of polymorphic sites in the diploid RHO sequences, all alleles were reconstructed using PHASE 2.1 [31] implemented in DNAsp 5.10.1 with default settings [32] (nuDNA hereafter). Only allelic states with probability higher than 70% were included in the proceeding analyses, as recommended by Stephens et al. [31]. The heterozygous sequences were deposited in GenBank with the degenerate bases following the IUPAC ambiguity code (S1 Table).

### Genetic diversity

Molecular diversity indices [number of polymorphic sites (S), haplotype number (H), percentage of private haplotypes (Hp), haplotype diversity (*h*), nucleotide diversity (π)] was assessed by measuring haplotype and nucleotide diversity. Changes in population size were tested using Fu’s Fs [33] and Tajima’s D [34]. We only considered expansion events congruent by both tests in order to avoid false positives driven by higher sensitivity of Fu’s Fs [35]. Genetic diversity indices were also estimated for each population (i.e. genetic cluster) identified by Geneland, as well as for coastal (RN+BA) and oceanic island localities (SS+FE+RA). Isolation by distance was tested using the Mantel test. All tests were performed in Arlequin 3.5 [36]. Demography history was also tested using a Bayasian Skyline Plot [37] implemented in BEAST software [38].

### Population structure

The genetic structure among the sampling sites of *Entomacrodus vomerinus* was assessed using the mitochondrial and nuclear genes separately through the fixation index F_ST_, between all pairwise comparisons (at a significance level α = 0.05). Three competing hypotheses of population structure were tested using the analysis of molecular variance (AMOVA): 1) an hypothesis contrasting insular against coastal habitats that is congruent with morphological differentiation (SS+FE+RA / RN+BA); 2) an hypothesis based on the four ecoregions sampled (SS / FE+RA / RN / BA); and 3) an hypothesis based on the three ecoregions (SS / FE+RA / RN+BA). This last hypothesis considers the coastal sampled area as the same ecoregion because localities in BA are situated in the border of two ecoregions. Those tests were performed in Arlequin 3.5 [36].

Haplotype networks were obtained for mtDNA and nuDNA data with PopART 1.7 [39], in order to observe the overall patterns of genealogies. Genetic structure was finally assessed in mitochondrial and nuclear genes combined, using the Bayesian assignment test implemented in Geneland 4.0.3 [40, 41]. This multilocus analysis is able to use haploid and diploid sequencing data to make an a posterior estimate of the most likely number of genetic clusters (K), while assigning individuals to those clusters in a spatially explicit manner that allows the identification of genetic breaks along the sampled area. Through a Markov chain Monte Carlo (MCMC) method, with nine replicates (5 x 10^6^ iterations in each) of K from 1 to 10 was used to determine the most probable number of populations (or clusters).

### Demographic parameters

Demographic parameters were estimated using the multilocus coalescent method implemented in IMa2 [42]. In this ‘isolation with migration model’, at certain time (t) an ancestor population with population size Ne_A_ splits into two extant populations that may differ in population size (Ne_1_ and Ne_2_), and where migration in both directions (m_1_ and m_2_) may occur after divergence. Comparing this 6-parameter model to a simpler 4-parameter model without migration, a likelihood ratio test can be used to test for ‘migration’ versus ‘isolation’ between descendent taxa [43]. This approach was applied to adjacent pairs of populations, as identified above, in order to test for differences in effective population size of the extant populations (Ne), and differences in population migration rates (2Nm).

Because this method assumes no intragenic recombination, this assumption was tested on phased data using the PhiPack software [44], with a window size of 100 bp and α = 0.05.

## Results

### Genetic diversity

Sequences of COI of 544 bp were obtained from 72 individuals of *Entomacrodus vomerinus*. A total of 15 sites were polymorphic (seven parsimony informative), resulting in 12 haplotypes. CYTB yielded 580 bp sequences from 71 individuals, with 17 polymorphic sites (ten parsimony informative) in 15 haplotypes. Concatenated mitochondrial data included 1,124 bp from 65 individuals with 29 polymorphic sites in 18 haplotypes.

RHO resulted in 441bp sequences from 83 individuals, and eight polymorphic sites. PHASE analysis recovered the alleles of 81 diploid sequences (i.e. 162 haplotypes used in the analyses). Two individuals from BA were removed from the analysis because their likelihood probabilities of the estimated allele sequences were lower than 70%. Nine alleles were identified with six polymorphic sites (five parsimony informative) (Table 1).

**Table 1:** Molecular parameters of *Entomacrodus vomerinus* in coastal and oceanic islands of the Tropical Southwestern Atlantic province. MtDNA includes 1,124 base pairs (544 bp COI and 580 bp CYTB). NuDNA includes 441 bp (RHO). Location acronyms are provided in Fig 1.

Data	Location	N	H	%Hp	S	*h*	π	Fu’s Fs	Tajima’s D
mtDNA	Total	65	18	72%	29	0.876	0.003	-3.683	-1.109
	SS (North)	9	2	50%	7	0.500	0.003	5.672	1.601
	FE	16	7	43%	11	0.750	0.001	-2.226	-1.840*
	RA	10	5	20%	8	0.866	0.001	-0.362	-0.924
	RN	14	8	50%	13	0.868	0.003	-1.144	-0.194
	BA	16	8	50%	13	0.841	0.003	-1.002	-0.403
	Central (FE+RA)	26	8	62%	12	0.793	0.001	-1.799	-1.357
	Islands (SS+FE+RA)	35	9	66%	17	0.801	0.003	0.087	-0.597
	Coast (RN+BA)	30	12	75%	18	0.841	0.003	-2.296	-0.689
nuDNA	Total	162	9	44%	6	0.440	0.001	-4.991	-0.934
	SS (North)	28	3	33%	2	0.648	0.001	0.952	1.084
	FE	40	3	33%	2	0.337	0.000	-0.474	-0.498
	RA	20	3	33%	2	0.352	0.000	-0.774	-0.801
	RN	36	6	17%	4	0.393	0.001	-3.217*	-1.002
	BA	38	5	0%	4	0.247	0.000	-3.238*	-1.510*
	Central (FE+RA)	60	4	50%	3	0.345	0.000	-1.440	-0.858
	Islands (SS+FE+RA)	88	5	60%	3	0.509	0.001	-1.000	0.052
	Coast (RN+BA)	72	6	67%	4	0.316	0.001	-3.161*	-0.928

N—total number of individuals analyzed in each location.

H—number of haplotypes.

% Hp—percentage of private haplotypes.

S—number of polymorphic sites.

*h*—haplotype diversity.

π—nucleotide diversity.

Significant P values are marked with * (≤ 0.05).

The number of haplotypes was higher in the coastal area (12 and 6 for mtDNA and nuDNA, respectively) relative to the oceanic islands (9 and 5). The majority of haplotypes (72%) recovered from mtDNA were exclusive of a certain locality, although they were mainly singletons. Coastal habitats presented more private haplotypes (75% and 67%, for mtDNA and nuDNA) than the insular habitats (66% and 60%).

In mtDNA, haplotype diversity showed very similar values at both continental and island habitats, while the diversity of nuDNA was higher in the insular habitats (Table 1).

Statistical significance of both neutrality tests suggested a population expansion event in BA (indicated by nuDNA). Bayesian Skyline Plot indicated no change in demography history (S1 Fig).

The Mantel test showed no correlation between geographic and genetic distances (F_ST_) in the data from the three genetic markers (mtDNA: r = 0.359, P = 0.274; nuDNA: r = 0.450, P = 0.215).

### Population structure

The pairwise F_ST_ showed values ranging from -0.02 to 0.54 for the mtDNA and from -0.01 to 0.43 for nuDNA (Table 2). Both mtDNA and nuDNA indicated significant F_ST_ between most pairwise comparisons (80%), except between the closest oceanic islands (FE and RA) and between the coastal sites (RN and BA). Genetic differentiation was strongest between SS and all other localities.

**Table 2:** Fixation paired indices of F_ST_ between coastal and oceanic islands localities of *Entomacrodus vomerinus*.

		SS	FE	RA	RN
mtDNA	FE	0.548*			
	RA	0.493*	-0.020		
	RN	0.403*	0.294*	0.228*	
	BA	0.417*	0.315*	0.266*	-0.027
nuDNA	FE	0.310*			
	RA	0.348*	0.067		
	RN	0.386*	0.077*	0.059*	
	BA	0.430*	0.062*	0.049*	-0.015

Significant P values are marked with * (≤ 0.05).

The three scenarios tested with the AMOVA were not significant (S2 Table) for either mtDNA or nuDNA. However, the third scenario based on three ecoregions (SS / FE+RA / RN+BA) presented the highest values of F_CT_ (0.38 and 0.26 for mtDNA and nuDNA, respectively), and P values marginally significant (0.07 and 0.06).

The haplotype network of mtDNA data shows a high number of haplotypes shared between FE and RA individuals as well as between RN and BA, and a few shared with the other localities. For mtDNA, only one haplotype (hap1) was shared among all localities. Moreover, two haplotypes (hap2 and hap4) were found in all localities except in SS, also evidencing low genetic diversity in SS, with only two haplotypes, including one exclusive that is several mutational steps distant from the others The nuDNA showed a very similar scenario, but the allele diversity was lower. There were two alleles (hap1 and hap2) shared among all localities, three alleles shared by coastal specimens (hap4, hap5 and hap6) and four alleles exclusive for a certain locality (hap3 [RA], hap7 [FE], hap8 [RN] and hap9 [SS]) (Fig 1b).

**Fig 2:**
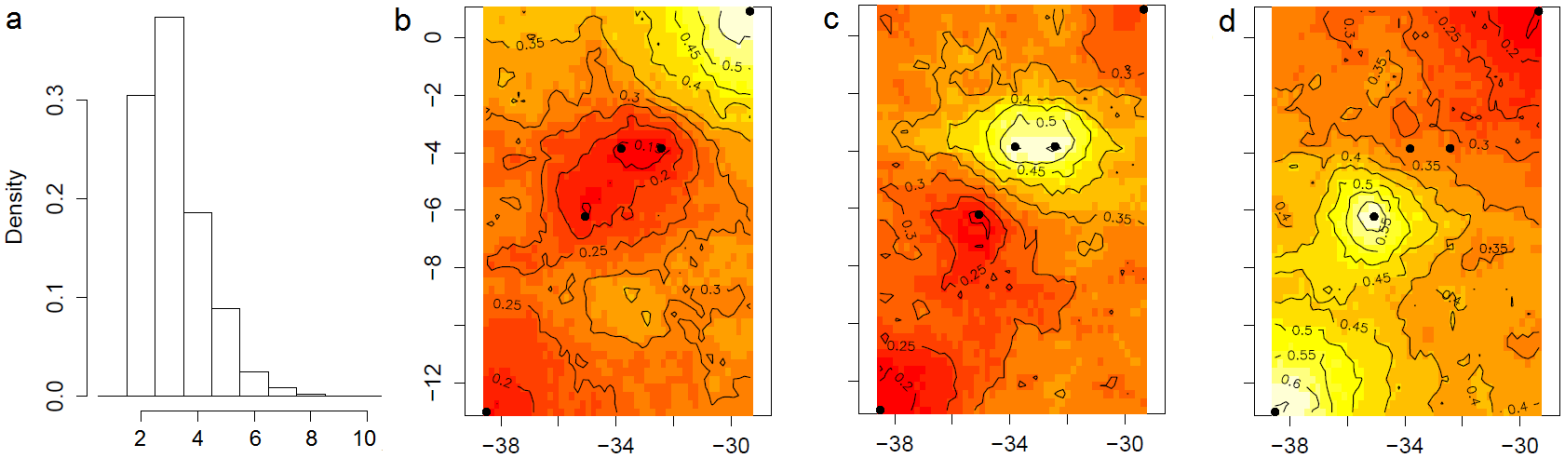
Geneland results showing the higher a posteriori probability of *Entomacrodus vomerinus* being partitioned in three populations. a Isoclines are depicted in each map indicating the posterior probabilities of a group of localities (black dots) belonging to the same population or genetic cluster (lighter zones). b The North population is composed by the São Pedro and São Paulo islands. c Central population by the Fernando de Noronha and Rocas Atoll. d Coastal population by Rio Grande do Norte and Bahia. Numbers on x and y-axes are longitude and latitude coordinates, respectively.

Geneland corroborates the F_ST_ results, suggesting three genetically different groups. K = 3 has the highest posterior probability in all the nine runs (33% to 38.5%), followed by K = 2 and K = 4 (Fig 2a). One genetic cluster comprises sampling localities from both coastal ecoregions (hereafter Coastal), another is composed by FE and RA ecoregion (hereafter Central), and the last one by SS ecoregion (hereafter North). (Fig 2b–2d).

### Demographic parameters

Based on the results above showing three population clusters, the ‘population with migration models’ were applied to a first comparison involving the North (SS) and Central populations (FE+RA), and to a second comparison involving the Central (FE+RA) and Coastal (RN+BA) populations. There was no significant signal of intragenic recombination. The estimation of demographic parameters reached very high values of ESS and consistent estimates across all runs, irrespective of the priors, indicating a good sampling of the parameter space and convergence in the same result.

**Fig 3:**
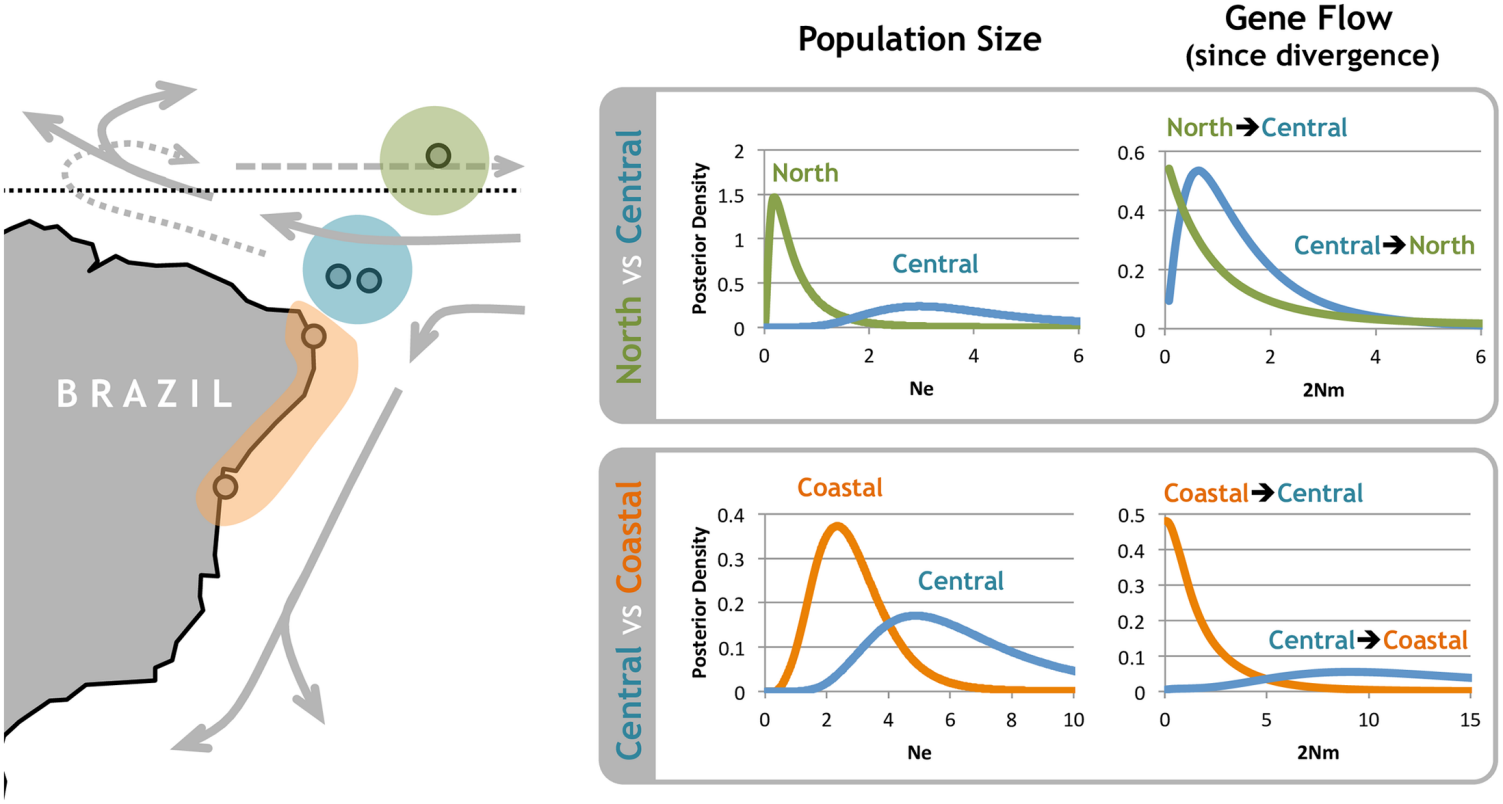
Estimation of demographic parameters for the three ESUs identified within *Entomacrodus vomerinus*. The Tropical Southwestern Atlantic map highlights: the sampling localities (circles), population clusters defining ESUs (colored polygons), ocean currents affecting those localities (full arrow—South-Equatorial current which splits in the Brazil Current and the North Brazil Current represented by the dotted arrow; dashed arrow—Equatorial Undercurrent [45]). The marginal posterior density curves represent the likelihood surface for the demographic parameters co-estimated in each of the two comparisons. Note that gene flow reflects 2Nm estimates forward in time, from population splitting to the present. The direction of migration when thinking forwards in time is reversed from that reflected by the 2Nm parameter [42] (Table 3), which is estimated backwards in time in the coalescent direction.

For both comparisons, there were marked differences in effective population size (Ne) and population migration rates (2Nm) (Table 3). In the North vs Central comparison, the estimated Ne was larger for the Central population, with the most likely value being outside of the 95% confidence interval estimated for the North population. Regarding population migration rates, as estimated backwards in time during the coalescent, 2Nm estimates are highly asymmetric. Population migration is significantly different from zero from North to Central (P < 0.01), but migration from Central to North could not be excluded. When interpreted forward in time (Fig 3), this means that since population splitting, gene flow has been occurring from Central into the North population, but not the other way around [42]. Likewise, in the Central vs Coastal comparison, the estimated Ne was larger for the Central population, and gene flow was significantly asymmetric (P < 0.05) from the Central into Coastal. In summary, these results show that in terms of genetic diversity, the Central population is larger and acts as source of gene flow, while the other two populations are smaller and act as sinks.

**Table 3:** Estimation of effective population size (Ne) and gene flow (population migration rates, 2Nm) among the three populations (ESUs) of *Entomacrodus vomerinus* from the Brazilian oceanic islands (North and Central) and coast (Coastal).

Comparison between ESUs (Pop. 1 vs 2)	Population Size		Population Migration (backwards in time)			
	Pop. 1	Pop. 2	From 1 into 2	LLR test	From 2 into 1	LLR test
North vs Central	0.188 (0.023–1.792)	2.962 (1.042–11.83)	2.650 (0.15–17.25)	7.410**	0.01 (0.0–8.5)	0.000ns
Central vs Coastal	2.348 (0.743–5.258)	4.883 (2.078–12.4)	0.008 (0.0–6.79)	0.013ns	0.25 (0.25–11.14)	4.449*

Estimated values report highest value in the histogram of probability densities (HiPt), followed by 95% HPD confidence intervals; LLR test refer to Likelihood ratio test [43]; Statistical significance is indicated by asterisks (*P< 0.05; **P< 0.01), or lack thereof by ‘ns’; The direction of migration is estimated during the coalescent and therefore backwards in time, i.e. from the present to population split.

## Discussion

### Genetic structure congruent with marine ecoregions

Previous morphological study pointed out differentiation between the oceanic (SS, FE) and coastal individuals of *Entomacrodus vomerinus* [13], suggesting that geographic and/or ecological isolation between the insular and coastal environments were associated to divergence within this species [11,17].

When testing the morphological hypothesis using an AMOVA, we did not find support for insular and coastal groups, suggesting either low statistical power based on single locus analysis or that a different population structure approach could better explain the patterns of genetic differentiation in *E*. *vomerinus*. However, small sampling sizes could also result in low statistical power of the AMOVA analysis to detect population differences [46].

Instead, our single locus approach (F_ST_) based on mtDNA and nuDNA data was more consistent with evolutionary divergence within *E*. *vomerinus* in three distinct populations (SS / FE+RA / RN+BA). In fact, when using the multilocus assignment test implemented in Geneland (Fig 2) we found strong statistical support for three genetic clusters, or populations, within the species’ range: Coastal (RN+BA), Central (FE+RA) and North (SS), although the probability of two clusters was also high (Fig 2a).

The spatial assignment of individual multilocus genotypes to the three inferred genetic clusters allowed identifying geographic and ecological barriers that can be associated with the divergence process. The three genetic clusters found in *E*. *vomerinus* are strongly associated with three ecoregions, marked by profound topographic and ecologic differences that might have driven evolutionary diversification. These marine ecoregions are cohesive ecological units that are likely to affect the most sedentary species, via geographic isolation, upwelling, nutrient inputs, freshwater influx, temperature regimes, sediments, currents, and bathymetric or coastal complexity [11]. Given that the Mantel tests showed that the distribution of gene frequencies over the species range does not follow a simple pattern of isolation by distance, this raises the hypothesis that isolation by environment (IBE) [47]. In the literature, IBE is a frequent pattern of population isolation shaping the patterns of genetic differentiation and gene flow in marine fishes [48,49]. This hypothesis can now be addressed in *E*. *vomerinus*.

In agreement with the genetic differentiation of *E*. *vomerinus* in three populations coincident with ecoregions, F_ST_ shows significant genetic differentiation between most pairwise comparisons (Table 2). The exceptions were the two coastal localities (BA+RN) and the two geographically close islands (FE+RA), where each pair of localities seems to share the same gene pool and similar environments. Thus, genetic similarity found between these locations can be explained due to environmental connectivity, as the Brazilian reef area extends for about 3000 km along the continental shelf [50], encompassing RN and BA. Similarly, FE and RA are part of the same seamount chain that extends from the coast of Ceará state [51]. In general, genetic breaks in tide pool fishes are often congruent with geographical isolation, oceanic currents, duration of larvae development, and factors that affect larvae settlement [48,52]. All of these factors may contribute to shape the observed structure of the studied species.

Notably, the North population (SS) was always the most differentiated locality both in mtDNA and nuDNA, suggesting that it might be genetically isolated from the remaining populations. This ecoregion (SS) seems to be a result of a young active tectonism and are composed by very rugged scarps with very few rockpools [53]. It is located at north of the equator, 1,000 km from the northeastern coast of Brazil and 1,800 km from the African coast [54]. Fish species from SS and believed to colonize this ecoregion from the closest habitat at FE island, leading Joyeux et al. [55] to consider SS as a "FE impoverished caricature". The genetic pattern found in *E*. *vomerinus* suggests that the SS population might also result from a relatively recent colonization from the closest Central population (FE+RA).

Similar phylogeographic breaks to those reported here in *E*. *vomerinus*, particularly the break separating the North group, were also detected in other reef fish species, such as *Chromis multilineata* (Pomacentridae) and *Caranx lugrubis* (Carangidae) [56,57], suggesting that these phylogeographic barriers might work as a useful biogeographic hypothesis for co-distributed species. Together, our results suggest significant evolutionary diversification within *E*. *vomerinus*. The three genetic clusters inhabit geographically and ecological distinct regions. Therefore, oceanographic and ecologic features have resulted in three Evolutionary Significant Units (ESUs) that need to be taken into consideration when accessing current population dynamics and conservation efforts.

### Genetic connectivity suggests a source-sink dynamics

Genetic diversity might not be equally partitioned among populations due to important demographic processes that occur after divergence, such as differences in effective population size or differences in the amount and direction of gene flow. Using coalescent methods (Table 3, Fig 3), we were able to estimate such parameters for the three ESUs found in *Entomacrodus vomerinus*.

Effective population size (Ne) was notably different between ESUs. Both in North vs Central and in the Central vs Coastal comparisons, the Central ESU always showed to be much larger than the other ESUs. This suggests that, irrespective of the census size of each population (N), the number of reproducing individuals contributing to the gene pool (Ne) is larger in the Central ESU. This can be explained by differences in habitat suitability and species abundance, which is known to strongly vary along the species distribution. In the coast, *E*. *vomerinus* has a relatively low abundance, 0.07 fishes/20 m^2^ [58], which may result from habitat competition with other reef fish or due to anthropogenic activities (e.g. pollution, tourism pressure) related to the intense urban occupation [59]. In the Central ESU, the species abundance is notoriously high, 10–25 fish/20 m^2^ in FE island [60]. In fact, islands are acknowledged for their higher abundance and lower species richness when compared to continental areas [61] and it can explain the higher density found there. Finally, at the North ESU there is a low relative abundance of *E*. *vomerinus*, which can be explained by the small suitable intertidal area for the species.

Population migration has also shown to be highly asymmetric. In both pairwise comparisons, the Central ESU does not receive gene flow either from the North or from the Coast ESU. Nevertheless, there is significant gene flow from the Central ESU into the others (Fig 3), suggesting a strong source-sink population dynamics. These results are in agreement with the life history traits and the surface circulation pattern of this region that determine dispersal potential of *E*. *vomerinus*. Since adults are sedentary and criptobentic, the connectivity between populations probably occurs mainly by larval transport [6], which are highly dependent of oceanographic currents. The South-Equatorial current moves westwards in the south Atlantic, in the direction of the islands forming the Central ESU. Around this area, this current splits into the North Brazil Current, which moves to the northern hemisphere, and into the Brazil Current, that flows southwards along the coast of Brazil [62] and where the Coastal ESU is located. Additionally, the Equatorial Undercurrent [45,63] moves eastwards towards Africa, passing north the Equator through the islands constituting the North ESU (Fig 3).

Although the duration of the larval period in *Entomacrodus* is unknown, this period in other species of the Blenniidae family varies between 50 days (*Ophioblennius atlanticus* [62]) and 29 days (*Lipophrys pholis* [64]). Thus, given the speed of these currents, a pelagic larva can be passively carried from the shore of Brazil to the North ESU in approximately three weeks [45], and from the Central ESU to the Coastal ESU in shorter time. Most importantly, these oceanographic currents make it difficult for pelagic organisms to circulate in the reverse directions, setting up the conditions for asymmetric dispersal and justifying the absence of gene flow into the Central ESU (Table 3). This finding of asymmetric dispersion has been previously documented in other species [65,66].

Together, these results suggest that the Central ESU, occupying the Fernando de Noronha and Rocas Atoll, has a key role in the genetic connectivity among populations of *E*. *vomerinus*. Most importantly, our results show that it acts as an important source of gene flow, while the remaining populations act as sinks, highlighting the importance of this ESU for the evolutionary dynamics in this species.

### Implications for conservation

Evolutionarily Significant Unit (ESU) is a qualitative criteria based on the distribution of population genetic diversity and is used as a basis for species conservation efforts [10]. *Entomacrodus vomerinus* showed three ESUs based on mtDNA and nuDNA data, North (SS), Central (FE+RA) and Coastal (RN+BA). Of those, three oceanic archipelagos (representing North and Central ESUs) are in marine protected areas (MPAs): the Rocas Atoll Biological Reserve (RA), Fernando de Noronha Marine National Park (FE) and Fernando de Noronha Environmental Protected Area (SS, FE and RA). The Central ESU is the main source of gene flow to the Coastal and North ESUs.

This result corroborates the importance of these Central oceanic islands MPAs for the maintenance of *E*. *vomerinus* genetic diversity along the Brazilian coast. Even though this species is not currently threatened, it is essential to conserve their shallow reefs habitats that are under different human-mediated pressures, ensuring the preservation of genetic variability of the tidepool communities, including some endemic species, mainly in the oceanic islands [51]. Furthermore, the pattern observed in *E*. *vomerinus* may reflect more general biogeographic patterns, reinforcing its importance as an indicator species for these ecoregions.

Greater efforts should be targeted to population bioecological assessments on the Brazilian coast in order to understanding the low density in this region, which also reflects is lower genetic variability and effective population size. Future efforts may be able to test if the observed low genetic diversity is due to naturally small populations limited by competition with other species, or if the species respond to human-mediated coastal impacts.

## Supporting Information

S1 Fig Bayesian Skyline Plot of the North ESU (SS), Central ESU (FE+RA) and Coastal ESU (RN+BA) of *Entomacrodus vomerinus* individuals. (TIF)

S1 Table *Entomacrodus vomerinus* specimens included in this study, sampling site, geographic coordinates and sequence accession number. (DOCX)

S2 Table Analysis of Molecular Variance (AMOVA) of *Entomacrodus vomerinus*. (DOCX)
