# Supplementary figures and images for: Population Structure of the Rockpool Blenny Entomacrodus vomerinus Shows Source-Sink Dynamics among Ecoregions in the Tropical Southwestern Atlantic
Source: PLoS One. 2016 Jun 16;11(6):e0157472. doi: 10.1371/journal.pone.0157472 (PMC4910989; doi:10.1371/journal.pone.0157472)

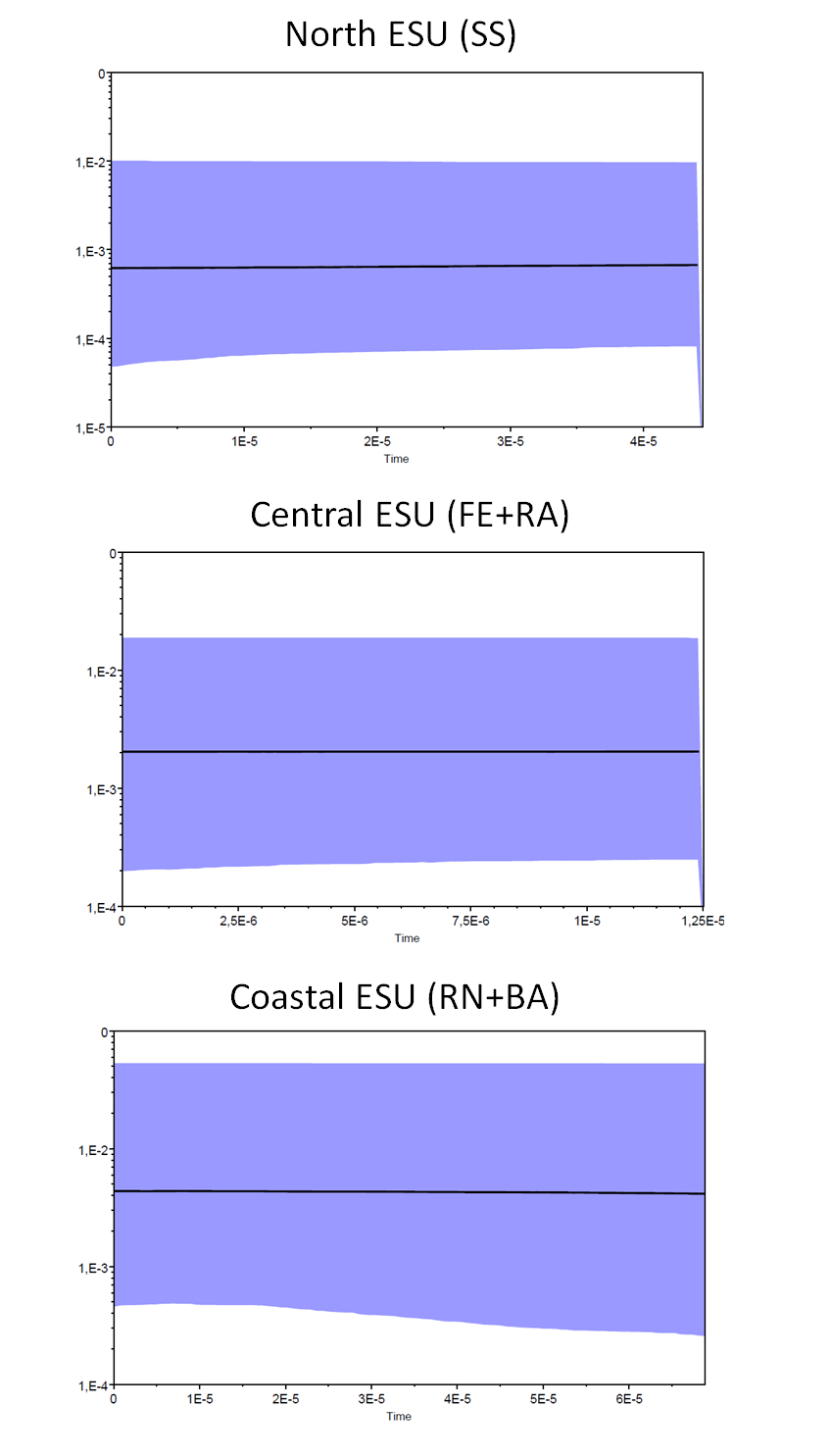

Supplement: S1 Fig — (TIF) [file pone.0157472.s001.tif]
