# Supplementary material for: Population Structure of the Rockpool Blenny Entomacrodus vomerinus Shows Source-Sink Dynamics among Ecoregions in the Tropical Southwestern Atlantic
Source: PLoS One. 2016 Jun 16;11(6):e0157472. doi: 10.1371/journal.pone.0157472 (PMC4910989; doi:10.1371/journal.pone.0157472)
Supplement: S1 Table — (DOCX) [file pone.0157472.s002.docx]

S1 Table. *Entomacrodus vomerinus* specimens included in this study, sampling site, geographic coordinates and sequence accession number. BA, Bahia State; PE, Pernambuco State; RN, Rio Grande do Norte State UFRN, Universidade Federal do Rio Grande do Norte.

| Catalogue  number | Specimen | Sampling site | Latitude | Longitude | GenBank accession number | | |
| --- | --- | --- | --- | --- | --- | --- | --- |
|  |  |  |  |  | COI | CYTB | RHO |
| UFRN1289 | SS1 | Arquipélago de São Pedro e São Paulo, PE, Brazil | 00º55’00.7”N | 29º20’44.9”W | *-* | KP714844 | *-* |
| UFRN1289 | SS2 | Arquipélago de São Pedro e São Paulo, PE, Brazil | 00º55’00.7”N | 29º20’44.9”W | *-* | KP714845 | KP715031 |
| UFRN1289 | SS3 | Arquipélago de São Pedro e São Paulo, PE, Brazil | 00º55’00.7”N | 29º20’44.9”W | KP714935 | *-* | *-* |
| UFRN1289 | SS4 | Arquipélago de São Pedro e São Paulo, PE, Brazil | 00º55’00.7”N | 29º20’44.9”W | KP714936 | KP714846 | KP715032 |
| UFRN1289 | SS5 | Arquipélago de São Pedro e São Paulo, PE, Brazil | 00º55’00.7”N | 29º20’44.9”W | KP714937 | KP714847 | KP715033 |
| UFRN1289 | SS6 | Arquipélago de São Pedro e São Paulo, PE, Brazil | 00º55’00.7”N | 29º20’44.9”W | *-* | KP714848 | KP715034 |
| UFRN1289 | SS7 | Arquipélago de São Pedro e São Paulo, PE, Brazil | 00º55’00.7”N | 29º20’44.9”W | *-* | KP714849 | KP715035 |
| UFRN1289 | SS8 | Arquipélago de São Pedro e São Paulo, PE, Brazil | 00º55’00.7”N | 29º20’44.9”W | KP714938 | KP714850 | KP715036 |
| UFRN1289 | SS9 | Arquipélago de São Pedro e São Paulo, PE, Brazil | 00º55’00.7”N | 29º20’44.9”W | KP714939 | *-* | KP715037 |
| UFRN1289 | SS10 | Arquipélago de São Pedro e São Paulo, PE, Brazil | 00º55’00.7”N | 29º20’44.9”W | *-* | KP714851 | KP715038 |
| UFRN1289 | SS11 | Arquipélago de São Pedro e São Paulo, PE, Brazil | 00º55’00.7”N | 29º20’44.9”W | KP714940 | KP714852 | KP715039 |
| UFRN1289 | SS12 | Arquipélago de São Pedro e São Paulo, PE, Brazil | 00º55’00.7”N | 29º20’44.9”W | KP714941 | KP714853 | KP715040 |
| UFRN1289 | SS13 | Arquipélago de São Pedro e São Paulo, PE, Brazil | 00º55’00.7”N | 29º20’44.9”W | KP714942 | KP714854 | KP715041 |
| UFRN1289 | SS14 | Arquipélago de São Pedro e São Paulo, PE, Brazil | 00º55’00.7”N | 29º20’44.9”W | KP714943 | KP714855 | KP715042 |
| UFRN1289 | SS15 | Arquipélago de São Pedro e São Paulo, PE, Brazil | 00º55’00.7”N | 29º20’44.9”W | KP714944 | KP714856 | KP715043 |
| UFRN1290 | SS16 | Arquipélago de São Pedro e São Paulo, PE, Brazil | 00º55’00.7”N | 29º20’44.9”W | KP714945 | KP714857 | KP715044 |
| UFRN058 | FE1 | Arquipélago de Fernando de Noronha, PE, Brazil | 03º50’38.5”S | 32º25’43.3”W | KP714900 | KP714813 | KP714993 |
| UFRN058 | FE2 | Arquipélago de Fernando de Noronha, PE, Brazil | 03º50’38.5”S | 32º25’43.3”W | KP714901 | KP714814 | KP714994 |
| UFRN058 | FE3 | Arquipélago de Fernando de Noronha, PE, Brazil | 03º50’38.5”S | 32º25’43.3”W | KP714902 | KP714815 | KP714995 |
| UFRN058 | FE4 | Arquipélago de Fernando de Noronha, PE, Brazil | 03º50’38.5”S | 32º25’43.3”W | KP714903 | KP714816 | KP714996 |
| UFRN058 | FE5 | Arquipélago de Fernando de Noronha, PE, Brazil | 03º50’38.5”S | 32º25’43.3”W | KP714904 | *-* | KP714997 |
| UFRN058 | FE6 | Arquipélago de Fernando de Noronha, PE, Brazil | 03º50’38.5”S | 32º25’43.3”W | KP714905 | KP714817 | KP714998 |
| UFRN058 | FE7 | Arquipélago de Fernando de Noronha, PE, Brazil | 03º50’38.5”S | 32º25’43.3”W | KP714906 | KP714818 | KP714999 |
| UFRN058 | FE8 | Arquipélago de Fernando de Noronha, PE, Brazil | 03º50’38.5”S | 32º25’43.3”W | KP714907 | *-* | KP715000 |
| UFRN058 | FE9 | Arquipélago de Fernando de Noronha, PE, Brazil | 03º50’38.5”S | 32º25’43.3”W | KP714908 | KP714819 | KP715001 |
| UFRN058 | FE10 | Arquipélago de Fernando de Noronha, PE, Brazil | 03º50’38.5”S | 32º25’43.3”W | KP714909 | KP714820 | KP715002 |
| UFRN058 | FE11 | Arquipélago de Fernando de Noronha, PE, Brazil | 03º50’38.5”S | 32º25’43.3”W | KP714910 | KP714821 | KP715003 |
| UFRN058 | FE12 | Arquipélago de Fernando de Noronha, PE, Brazil | 03º50’38.5”S | 32º25’43.3”W | *-* | *-* | KP715004 |
| UFRN058 | FE13 | Arquipélago de Fernando de Noronha, PE, Brazil | 03º50’38.5”S | 32º25’43.3”W | KP714911 | KP714822 | KP715005 |
| UFRN056 | FE14 | Arquipélago de Fernando de Noronha, PE, Brazil | 03º50’38.5”S | 32º25’43.3”W | KP714912 | *-* | KP715006 |
| UFRN056 | FE15 | Arquipélago de Fernando de Noronha, PE, Brazil | 03º50’38.5”S | 32º25’43.3”W | KP714913 | KP714823 | KP715007 |
| UFRN056 | FE16 | Arquipélago de Fernando de Noronha, PE, Brazil | 03º50’38.5”S | 32º25’43.3”W | KP714914 | KP714824 | KP715008 |
| UFRN056 | FE17 | Arquipélago de Fernando de Noronha, PE, Brazil | 03º50’38.5”S | 32º25’43.3”W | KP714915 | KP714825 | KP715009 |
| UFRN056 | FE18 | Arquipélago de Fernando de Noronha, PE, Brazil | 03º50’38.5”S | 32º25’43.3”W | KP714916 | KP714826 | KP715010 |
| UFRN056 | FE19 | Arquipélago de Fernando de Noronha, PE, Brazil | 03º50’38.5”S | 32º25’43.3”W | KP714917 | KP714827 | KP715011 |
| UFRN056 | FE20 | Arquipélago de Fernando de Noronha, PE, Brazil | 03º50’38.5”S | 32º25’43.3”W | KP714918 | KP714828 | KP715012 |
| UFRN1387 | RA11 | Falsa Barreta, Atol das Rocas, RN, Brazil | 03º52’19.2”S | 33º47’52.6”W | KP714874 | KP714787 | KP714962 |
| UFRN1387 | RA 12 | Falsa Barreta, Atol das Rocas, RN, Brazil | 03º52’19.2”S | 33º47’52.6”W | KP714875 | KP714788 | KP714963 |
| UFRN1387 | RA 13 | Falsa Barreta, Atol das Rocas, RN, Brazil | 03º52’19.2”S | 33º47’52.6”W | KP714876 | KP714789 | KP714964 |
| UFRN1387 | RA 14 | Falsa Barreta, Atol das Rocas, RN, Brazil | 03º52’19.2”S | 33º47’52.6”W | KP714877 | KP714790 | KP714965 |
| UFRN1387 | RA 15 | Falsa Barreta, Atol das Rocas, RN, Brazil | 03º52’19.2”S | 33º47’52.6”W | KP714878 | KP714791 | KP714966 |
| UFRN1387 | RA 16 | Falsa Barreta, Atol das Rocas, RN, Brazil | 03º52’19.2”S | 33º47’52.6”W | KP714879 | KP714792 | KP714967 |
| UFRN1387 | RA 17 | Falsa Barreta, Atol das Rocas, RN, Brazil | 03º52’19.2”S | 33º47’52.6”W | KP714880 | KP714793 | KP714968 |
| UFRN1387 | RA 18 | Falsa Barreta, Atol das Rocas, RN, Brazil | 03º52’19.2”S | 33º47’52.6”W | KP714881 | KP714794 | KP714969 |
| UFRN1387 | RA 19 | Falsa Barreta, Atol das Rocas, RN, Brazil | 03º52’19.2”S | 33º47’52.6”W | KP714882 | KP714795 | KP714970 |
| UFRN1387 | RA 20 | Falsa Barreta, Atol das Rocas, RN, Brazil | 03º52’19.2”S | 33º47’52.6”W | KP714883 | KP714796 | KP714971 |
| UFRN441 | RN1 | Tibau do Sul, RN, Brazil | 06º13’37.8”S | 35º03’05.2”W | KP714919 | KP714829 | KP715013 |
| UFRN441 | RN2 | Tibau do Sul, RN, Brazil | 06º13’37.8”S | 35º03’05.2”W | KP714920 | KP714830 | KP715014 |
| UFRN441 | RN3 | Tibau do Sul, RN, Brazil | 06º13’37.8”S | 35º03’05.2”W | *-* | *-* | KP715015 |
| UFRN441 | RN5 | Tibau do Sul, RN, Brazil | 06º13’37.8”S | 35º03’05.2”W | KP714921 | KP714831 | KP715016 |
| UFRN441 | RN6 | Tibau do Sul, RN, Brazil | 06º13’37.8”S | 35º03’05.2”W | KP714922 | KP714832 | KP715017 |
| UFRN441 | RN7 | Tibau do Sul, RN, Brazil | 06º13’37.8”S | 35º03’05.2”W | KP714923 | KP714833 | KP715018 |
| UFRN441 | RN8 | Tibau do Sul, RN, Brazil | 06º13’37.8”S | 35º03’05.2”W | KP714924 | KP714834 | KP715019 |
| UFRN441 | RN9 | Tibau do Sul, RN, Brazil | 06º13’37.8”S | 35º03’05.2”W | KP714925 | *-* | KP715020 |
| UFRN441 | RN10 | Tibau do Sul, RN, Brazil | 06º13’37.8”S | 35º03’05.2”W | KP714926 | KP714835 | KP715021 |
| UFRN441 | RN11 | Tibau do Sul, RN, Brazil | 06º13’37.8”S | 35º03’05.2”W | KP714927 | KP714836 | KP715022 |
| UFRN441 | RN12 | Tibau do Sul, RN, Brazil | 06º13’37.8”S | 35º03’05.2”W | KP714928 | KP714837 | KP715023 |
| UFRN441 | RN13 | Tibau do Sul, RN, Brazil | 06º13’37.8”S | 35º03’05.2”W | KP714929 | KP714838 | KP715024 |
| UFRN441 | RN14 | Tibau do Sul, RN, Brazil | 06º13’37.8”S | 35º03’05.2”W | KP714930 | KP714839 | KP715025 |
| UFRN441 | RN15 | Tibau do Sul, RN, Brazil | 06º13’37.8”S | 35º03’05.2”W | *-* | KP714840 | KP715026 |
| UFRN441 | RN16 | Tibau do Sul, RN, Brazil | 06º13’37.8”S | 35º03’05.2”W | KP714931 | KP714841 | KP715027 |
| UFRN441 | RN17 | Tibau do Sul, RN, Brazil | 06º13’37.8”S | 35º03’05.2”W | KP714932 | KP714842 | KP715028 |
| UFRN441 | RN18 | Tibau do Sul, RN, Brazil | 06º13’37.8”S | 35º03’05.2”W | KP714933 | KP714843 | KP715029 |
| UFRN441 | RN19 | Tibau do Sul, RN, Brazil | 06º13’37.8”S | 35º03’05.2”W | KP714934 | *-* | KP715030 |
| UFRN078 | BA1 | Praia de Ondina, Salvador, Bahia, Brazil | 13º00’40.8”S | 38º30’32.1”W | *-* | *-* | KP714972 |
| UFRN078 | BA2 | Praia de Ondina, Salvador, Bahia, Brazil | 13º00’40.8”S | 38º30’32.1”W | *-* | *-* | KP714973 |
| UFRN078 | BA3 | Praia de Ondina, Salvador, Bahia, Brazil | 13º00’40.8”S | 38º30’32.1”W | *-* | *-* | KP714974 |
| UFRN079 | BA4 | Praia de Ondina, Salvador, Bahia, Brazil | 13º00’40.8”S | 38º30’32.1”W | *-* | - | KP714975 |
| UFRN079 | BA5 | Praia de Ondina, Salvador, Bahia, Brazil | 13º00’40.8”S | 38º30’32.1”W | KP714884 | KP714797 | KP714976 |
| UFRN2519 | BA6 | Praia do Farol da Barra, Salvador, Bahia, Brazil | 13º00’14.8”S | 38º32’01.7”W | *-* | *-* | KP714977 |
| UFRN2519 | BA7 | Praia do Farol da Barra, Salvador, Bahia, Brazil | 13º00’14.8”S | 38º32’01.7”W | KP714885 | KP714798 | KP714978 |
| UFRN2519 | BA8 | Praia do Farol da Barra, Salvador, Bahia, Brazil | 13º00’14.8”S | 38º32’01.7”W | KP714886 | KP714799 | KP714979 |
| UFRN2519 | BA9 | Praia do Farol da Barra, Salvador, Bahia, Brazil | 13º00’14.8”S | 38º32’01.7”W | KP714887 | KP714800 | KP714980 |
| UFRN2519 | BA10 | Praia do Farol da Barra, Salvador, Bahia, Brazil | 13º00’14.8”S | 38º32’01.7”W | KP714888 | KP714801 | KP714981 |
| UFRN2519 | BA11 | Praia do Farol da Barra, Salvador, Bahia, Brazil | 13º00’14.8”S | 38º32’01.7”W | KP714889 | KP714802 | KP714982 |
| UFRN2519 | BA12 | Praia do Farol da Barra, Salvador, Bahia, Brazil | 13º00’14.8”S | 38º32’01.7”W | KP714890 | KP714803 | KP714983 |
| UFRN2519 | BA13 | Praia do Farol da Barra, Salvador, Bahia, Brazil | 13º00’14.8”S | 38º32’01.7”W | KP714891 | KP714804 | KP714984 |
| UFRN2519 | BA14 | Praia do Farol da Barra, Salvador, Bahia, Brazil | 13º00’14.8”S | 38º32’01.7”W | KP714892 | KP714805 | KP714985 |
| UFRN2519 | BA15 | Praia do Farol da Barra, Salvador, Bahia, Brazil | 13º00’14.8”S | 38º32’01.7”W | KP714893 | KP714806 | KP714986 |
| UFRN2519 | BA16 | Praia do Farol da Barra, Salvador, Bahia, Brazil | 13º00’14.8”S | 38º32’01.7”W | KP714894 | KP714807 | KP714987 |
| UFRN2519 | BA17 | Praia do Farol da Barra, Salvador, Bahia, Brazil | 13º00’14.8”S | 38º32’01.7”W | KP714895 | KP714808 | KP714988 |
| UFRN2519 | BA18 | Praia do Farol da Barra, Salvador, Bahia, Brazil | 13º00’14.8”S | 38º32’01.7”W | KP714896 | KP714809 | KP714989 |
| UFRN2519 | BA19 | Praia do Farol da Barra, Salvador, Bahia, Brazil | 13º00’14.8”S | 38º32’01.7”W | KP714897 | KP714810 | KP714990 |
| UFRN2519 | BA20 | Praia do Farol da Barra, Salvador, Bahia, Brazil | 13º00’14.8”S | 38º32’01.7”W | KP714898 | KP714811 | KP714991 |
| UFRN2519 | BA21 | Praia do Farol da Barra, Salvador, Bahia, Brazil | 13º00’14.8”S | 38º32’01.7”W | KP714899 | KP714812 | KP714992 |
