# Supplementary material for: Population Structure of the Rockpool Blenny Entomacrodus vomerinus Shows Source-Sink Dynamics among Ecoregions in the Tropical Southwestern Atlantic
Source: PLoS One. 2016 Jun 16;11(6):e0157472. doi: 10.1371/journal.pone.0157472 (PMC4910989; doi:10.1371/journal.pone.0157472)
Supplement: S2 Table — (DOCX) [file pone.0157472.s003.docx]

S2 Table. Analysis of Molecular Variance (AMOVA) of *Entomacrodus vomerinus*.

mtDNA (COI+CYTB)

| Insular x Continental hypothesis (SS+FE+RA/RN+BA) | | | |
| --- | --- | --- | --- |
|  | Percentage of variation | Fixation indices | Significance tests  P value |
| Among groups | 13.21% | F_CT_ = 0.13205 | 0.19257±0.01213 |
| Among populations within groups | 20.89% | F_SC_ = 0.24067 | 0.00000±0.00000 |
| Within populations | 65.91% | F_ST_ = 0.34095 | 0.00000±0.00000 |
|  | | | |
| Ecoregion hypothesis (SS/FE+RA/RN/BA) | | | |
|  | Percentage of variation | Fixation indices | Significance tests  P value |
| Among groups | 35.90% | F_CT_ = 0.35900 | 0.10655±0.01401 |
| Among populations within groups | -2.81% | F_SC_ = -0.04383 | 0.64418±0.01398 |
| Within populations | 66.91% | F_ST_ = 0.33090 | 0.00000±0.00000 |
|  | | | |
| Ecoregion + Geographic distance hypothesis (SS/FE+RA/RN+BA) | | | |
|  | Percentage of variation | Fixation indices | Significance tests  P value |
| Among groups | 38.65% | F_CT_ = 0.38651 | 0.07527±0.00565 |
| Among populations within groups | -1.78% | F_SC_ = -0.02903 | 0.75660±0.01574 |
| Within populations | 63.13% | F_ST_ = 0.36870 | 0.06158±0.00651 |

nuDNA (RHO)

| Insular x Continental hypothesis (SS+FE+RA/RN+BA) | | | |
| --- | --- | --- | --- |
|  | Percentage of variation | Fixation indices | Significance tests  P value |
| Among groups | 6.97% | F_CT_ = 0.06972 | 0.30792±0.01406 |
| Among populations within groups | 17.59% | F_SC_ = 0.18950 | 0.00000±0.00000 |
| Within populations | 75.44% | F_ST_ = 0.24559 | 0.00000±0.00000 |
|  | | | |
| Ecoregion hypothesis (SS/FE+RA/RN/BA) | | | |
|  | Percentage of variation | Fixation indices | Significance tests  P value |
| Among groups | 20.43% | F_CT_ = 0.20428 | 0.41056±0.01714 |
| Among populations within groups | 3.35% | F_SC_ = 0.04207 | 0.05474±0.00603 |
| Within populations | 76.23% | F_ST_ = 0.23775 | 0.00000±0.00000 |
|  | | | |
| Ecoregion + Geographic distance hypothesis (SS/FE+RA/RN+BA) | | | |
|  | Percentage of variation | Fixation indices | Significance tests  P value |
| Among groups | 26.11% | F_CT_ = 0.26114 | 0.06256±0.00910 |
| Among populations within groups | 0.67% | F_SC_ = 0.00909 | 0.17889±0.01170 |
| Within populations | 73.21% | F_ST_ = 0.26786 | 0.00000±0.00000 |
